# Supplementary material for: C/EBPδ-induced epigenetic changes control the dynamic gene transcription of S100a8 and S100a9
Source: eLife. 2022 May 11;11:e75594. doi: 10.7554/eLife.75594 (PMC9122501; doi:10.7554/eLife.75594)
Supplement: Supplementary file 1. — (a) List of guides (stated in 5’–3’ orientation) for cloning into lentiCRISPR v2, related to Materials and methods. (b) List of primer (stated in 5’–3’ orientation) for amplifying GeCKO library and NGS, related to Materials and methods. (c) List of oligonucleotides (stated in 5’–3’ orientation, fw: forward, rv: reverse) for cloning steps to construct TRE_3xFlag-C/EBPδ vector, related to Materials and methods. (d) List of oligonucleotides (stated in 5’–3’ orientation, fw: forward, rv: reverse) for cloning steps to construct S100a8 and S100a9 reporter construct, related to Materials and methods. (e) List of oligonucleotides (stated in 5’–3’ orientation) for mutagenesis to disrupt specific sites within S100a8 and S100a9 reporter vectors, related to Materials and methods. (f) List of quantitative reverse transcription polymerase chain reaction (qRT-PCR) primer (in 5’–3’ orientation) used for qRT-PCR, related to Materials and methods. (g) List of chromatin immunoprecipitation (ChIP)-PCR primer (in 5’–3’ orientation) for S100a8 and S100a9 genomic locations, related to Materials and methods. [file elife-75594-supp1.docx]

Supplementary file 1a: List of guides (stated in 5’-3’ orientation) for cloning into lentiCRISPRv2, related to Methods

| Gene | Forward primer | Reverse primer |
| --- | --- | --- |
| *Phf8* | CACCGTTACCACTGCCCTAACTGTG | AAACCACAGTTAGGGCAGTGGTAA |
| *Csrp1* | CACCGCGCTGTCCCCGCTGTAGCC | AAACGGCTACAGCGGGGACAGCGC |
| *Hand1* | CACCCCCGGCGAGAAGAGGATTAA | AAACTTAATCCTCTTCTCGCCGGG |
| *Fbxw7* | CACCGTGTGGCAACCGCATAGTTAG | AAACCTAACTATGCGGTTGCCACAC |
| *Atf3* | CACCGTCCTCAAATACCAGTGACCC | AAACGGGTCACTGGTATTTGAGAC |
| *Stat3* | CACCGACAGGCATCGGGCCAGATC | AAACGATCGTGGCCCGATGCCTGTC |
| *Klf5* | CACCGTCTTGATCTGTGTTACGCA | AAACTGCGTAACACAGATCAAGAC |
| *Irf7* | CACCGCATTTCGGTCGTAGGGATC | AAACGATCCCTACGACCGAAATGC |
| *Cebpb* | CACCGTCCGCGCGCTTGCAGTCCG | AAACCAGGCGCGCGAACGTCAGGC |

Supplementary file 1b: List of primer (stated in 5’-3’ orientation) for amplifying GeCKO library and NGS, related to Methods

| Oligo | Sequence |
| --- | --- |
| fw-1 | AATGATACGGCGACCACCGAGATCTACACTCTTTCCCTACACGACGCTCTTCCGATCTTAAGTAGAGGCTTTATATATCTTGTGGAAAGGACGAAACACC |
| fw-2 | AATGATACGGCGACCACCGAGATCTACACTCTTTCCCTACACGACGCTCTTCCGATCTATCATGCTTAGCTTTATATATCTTGTGGAAAGGACGAAACACC |
| fw-3 | AATGATACGGCGACCACCGAGATCTACACTCTTTCCCTACACGACGCTCTTCCGATCTGATGCACATCTGCTTTATATATCTTGTGGAAAGGACGAAACACC |
| fw-4 | AATGATACGGCGACCACCGAGATCTACACTCTTTCCCTACACGACGCTCTTCCGATCTCGATTGCTCGACGCTTTATATATCTTGTGGAAAGGACGAAACACC |
| fw-5 | AATGATACGGCGACCACCGAGATCTACACTCTTTCCCTACACGACGCTCTTCCGATCTTCGATAGCAATTCGCTTTATATATCTTGTGGAAAGGACGAAACACC |
| fw-6 | AATGATACGGCGACCACCGAGATCTACACTCTTTCCCTACACGACGCTCTTCCGATCTATCGATAGTTGCTTGCTTTATATATCTTGTGGAAAGGACGAAACACC |
| fw-7 | AATGATACGGCGACCACCGAGATCTACACTCTTTCCCTACACGACGCTCTTCCGATCTGATCGATCCAGTTAGGCTTTATATATCTTGTGGAAAGGACGAAACACC |
| fw-8 | AATGATACGGCGACCACCGAGATCTACACTCTTTCCCTACACGACGCTCTTCCGATCTCGATCGATTTGAGCCTGCTTTATATATCTTGTGGAAAGGACGAAACACC |
| fw-9 | AATGATACGGCGACCACCGAGATCTACACTCTTTCCCTACACGACGCTCTTCCGATCTACGATCGATACACGATCGCTTTATATATCTTGTGGAAAGGACGAAACACC |
| fw-10 | AATGATACGGCGACCACCGAGATCTACACTCTTTCCCTACACGACGCTCTTCCGATCTTACGATCGATGGTCCAGAGCTTTATATATCTTGTGGAAAGGACGAAACAC |
| KO-rv-4 | CAAGCAGAAGACGGCATACGAGATATTCTAGGGTGACTGGAGTTCAGACGTGTGCTCTTCCGATCTCCGACTCGGTGCCACTTTTTCAA |

Supplementary file 1c: List of oligonucleotides (stated in 5’-3’ orientation, fw: forward, rv: reverse) for cloning steps to construct TRE_3xFlag-C/EBPδ vector, related to Methods

| Oligo | Sequence |
| --- | --- |
| Flag fw | CTAGACTGCCACCATGGACTACAAAGACCATGACGGTGATTATAAAGATCATGACATCGATTACAAGGATGACGATGACAAGAG |
| Flag rv | AATTCTCTTGTCATCGTCATCCTTGTAATCGATGTCATGATCTTTATAATCACCGTCATGGTCTTTGTAGTCCATGGTGGCAGT |
| *Nhe*I fw | AGAATTGGCTAGCGCCGCCACCATGGACTACAAAGACCATG |
| *Age*I rv | TGGTGGACCGGTTCTTACCGGCAGTCGGCG |

Supplementary file 1d: List of oligonucleotides (stated in 5’-3’ orientation, fw: forward, rv: reverse) for cloning steps to constructS*100a8* and *S100a9* reporter construct, related to Methods

| Oligo | Sequence |
| --- | --- |
| *Cla*I-*s100a8*prom fw | AAATCGATTGCAGCATATGAGAAATACTCCATAGACC |
| *Xba*I-*s100a8*prom rv | CTTCTAGACCGCCTTCACGAAAGATTTCCTTTCAACT |
| *Cla*I-*s100a9*prom fw | AAATCGATTGCAGGACAAAGTTTGGAGCTAAAATGAGA |
| *Xba*I-*s100a9*prom rv | CTTCTAGACCGCCTTAGGTGCTTTGTTGGCCATCTT |

Supplementary file 1e: List of oligonucleotides (stated in 5’-3’ orientation) for mutagenesis to disrupt specific sites within *S100a8* and *S100a9* reporter vectors, related to Methods

| Oligo | Forward primer | Reverse primer |
| --- | --- | --- |
| *S100a8* | | |
| Site 1 | CCACATGGACTTGGTAACAGAGGCTGTGGC | GCCACAGCCTCTGTTACCAAGTCCATGTGG |
| Site 2 | CTTCCCTTCCAGAGTTGCTCTGTTTCCTAACCAA | TTGGTTAGGAAACAGAGCAACTCTGGAAGGGAAG |
| Site 3 | CATAGCCCTACTTGTCTTGAAGACGGAGCTGACAA | TTGTCAGCTCCGTCTTCAAGACAAGTAGGGCTATG |
| *S100a9* | | |
| Site 1 | CATAACATTCCTCTCCATGAGCTACCACCTCTGA | TCAGAGGTGGTAGCTCATGGAGAGGAATGTTATG |
| Site 2 | CTGTATATTACAAGTCAGGACTGAAAGGTATCAAAGATGGCA | TGCCATCTTTGATACCTTTCAGTCCTGACTTGTAATATACAG |
| Site 3 | GAGCTGTGTGAACGCCCCACCCCAGC | GCTGGGGTGGGGCGTTCACACAGCTC |
| Site 4 | TACCACACTGCTCACCTGTTCTTCCCCGAG | CTCGGGGAAGAACAGGTGAGCAGTGTGGTA |

Supplementary file 1f: List of qRT-PCR primer (in 5’-3’ orientation) used for qRT-PCR, related to Methods

| Gene | Forward primer | Reverse primer |
| --- | --- | --- |
| *Gapdh* | CCCACTCTTCCACCTTCGATG | GTCCACCACCCTGTTGCTGTAG |
| *S100a8* | ATCACCATGCCCTCTACAAGAATG | GTCCAATTCTCTGAACAAGTTTTCG |
| *S100a9* | AAGCTGCATGAGAACAACCCA | CCCAGAACAAAGGCCATTGA |
| *Cebpd* | GAACCCGCGGCCTTCTA | TGTTGAAGAGGTCGGCGA |
| *Jmjd3* | CCCCCATTTCAGCTGACTAA | CTGGACCAAGGGGTGTGTT |
| *Ptx3* | CGAAATAGACAATGGACTTCATCC | CATCTGCGAGTTCTCCAGCAT |
| *Cd209a* | TCATCCTTGTCAAAGTCTACAA | GGCCACAGAGAAGAAGTAAC |
| *Msr1* | AGTGCTGTCTTCTTTACCAGC | GTGAGGAAGGGATGCTGTC |
| *Cd14* | TTTGCATCCTCCTGGTTTCTGA | GCTTTTACCCACTGAACCATCTTG |
| *Tnfa* | AGAAACACAAGATGCTGGGACAGT | CCTTTGCAGAACTCAGGAATGG |
| *Il6* | TGAGATCTACTCGGCAAACCTAGTG | CTTCGTAGAGAACAACATAAGTCAGAT ACC |
| *Inos* | CCTCATTGGCCAGCTGCTT | GGTCCGCAAGAGAGTGCTGTT |
| *Cd86* | GACATACAATGAGCGAGATCG | GGAAACGGAGTCAATGAAGATTTC |
| *Il1b* | TGTCTTGGCCGAGGACTAAGG | TGGGCTGGACTGTTTCTAATGC |
| *Il10* | GGGTTGCCAAGCCTTATCG | TCTCACCCAGGGAATTCAAATG |
| *Cd163* | TTGGAATGGGTGGACACAGA | GTTAGTGACAGCAGAGGCACT |

Supplementary file 1g: List of ChIP-qPCR primer (in 5’-3’ orientation) for *S100a8* and *S100a9* genomic locations, related to Methods

| Position | Forward primer | Reverse primer |
| --- | --- | --- |
| *S100a8* | | |
| *-580 to -311* | TCCTCCAAAGACTGTACCTAC | GGATCCATTGCTGATCCAATG |
| *-247 to -79* | CATGGACTTATTGCCATGCCCC | TCTTCCCTTCCAGAGTTGCCAC |
| *+2 to +252* | GTCTAGCAGAAGAGGGCAG | GAAAGGAAAAGTCGCAGAGG |
| *S100a9* | | |
| *-851 to -614* | AGACTATCAGAGAGGTACAAGG | TCTTCAGTGGTTCTCAGTGT |
| *-371 to -239* | CATGAGAAGGCTCAGACCATA | AGCCTACCTGCCATCTTTG |
| *-78 to +28* | TGACTGTCAGAGTTACCACAC | TAAGAGCAGTGTAAGCCCAG |
| *+70 to +278* | GGCCACTGTTAGGCAAGATA | AGCCCTCCTAGGTTGATCTAT |
